# Outcomes of extracorporeal membrane oxygenation in critically ill patients with sickle cell disease: a systematic review and meta-analysis

**DOI:** 10.1016/j.aicoj.2026.100194

**Published:** 2026-10-28

**Authors:** Laure Flurin, Elsa Vidal, Thibault Parent, Amélie Rollé

**Affiliations:** a Anesthesiology and Intensive Care Department, University Hospital of La Guadeloupe, France; b APHP, Service de Médecine Intensive Réanimation, Institut de Cardiologie, Hôpital Pitié-Salpêtrière, 75013 Paris, France; c Université Paris Cité and Université des Antilles, INSERM, BIGR, F-75015 Paris, France

**Keywords:** Extracorporeal membrane oxygenation, Sickle cell disease, Acute chest syndrome, Critical care, Intensive care units, Mortality, Systematic review

## Abstract

**Purpose:**

Extracorporeal membrane oxygenation (ECMO) may be considered as rescue therapy in patients with sickle cell disease (SCD) presenting with life-threatening cardiorespiratory failure, particularly acute chest syndrome (ACS). However, outcomes of ECMO in this population remain poorly defined, especially in adult populations. We aimed to synthesize available evidence on mortality and complications associated with ECMO use in adult and pediatric populations with SCD.

**Methods:**

We conducted a systematic review and meta-analysis of observational studies reporting ECMO use in patients with confirmed SCD. MEDLINE, EMBASE, PubMed, and the Cochrane Central Register of Controlled Trials were searched from January 2000 to January 2025. Risk of bias for included studies was assessed using the ROBINS-I tool. Random-effects meta-analysis of proportions was performed to estimate pooled mortality. Exploratory study-level meta-regression was used to examine associations between mortality and prespecified moderators, including age group, ECMO modality, and ACS as the indication for ECMO.

**Results:**

Seven retrospective studies including 252 patients were included in the meta-analysis. The pooled mortality estimate was 64.5% (95% CI, 39.3–86.0%), with heterogeneity across studies (I² = 80%). Mortality estimates appeared numerically higher in studies including predominantly adult populations, greater use of veno-arterial ECMO, and a higher proportion of patients with ACS at cannulation. Exploratory meta-regression suggested trends toward higher predicted mortality with increasing proportions of adult patients and ACS, and lower mortality with higher proportions of veno-venous ECMO. Reporting of ECMO-related complications was inconsistent, and hemolysis was systematically assessed in only one study.

**Discussion:**

ECMO use in patients with SCD is associated with high mortality, particularly in severe clinical contexts such as ACS. Substantial heterogeneity and methodological limitations preclude firm conclusions. These findings underscore the need for prospective, disease-specific registries to better define patient selection and management strategies.’

## Introduction

Sickle cell disease (SCD) is a severe autosomal recessive hemoglobinopathy affecting approximately 300,000 newborns worldwide each year [1,2]. It remains associated with substantial morbidity and premature mortality [3]. A single point mutation in the β-globin gene leads to the production of hemoglobin S, which polymerizes under hypoxic conditions, resulting in red blood cell sickling, hemolysis, and microvascular obstruction [4,5]. These mechanisms drive recurrent vaso-occlusive events and progressive multiorgan dysfunction, particularly involving the cardiopulmonary and renal systems [6]. Acute chest syndrome (ACS) represents the most severe pulmonary complication of SCD and is a leading cause of intensive care unit admission and early death [7]. Severe ACS may progress to acute respiratory distress syndrome, frequently complicated by pulmonary hypertension, right ventricular failure, and refractory hypoxemia [8]. In such life-threatening situations, extracorporeal membrane oxygenation (ECMO) may be considered as a rescue therapy [9]. The complex pathophysiology of ACS, the high prevalence of multiorgan dysfunction, and disease-specific management challenges—including transfusion strategies, anticoagulation, and hemolysis—raise concerns regarding both the feasibility and outcomes of ECMO in this population. Therefore, the role of ECMO in patients with SCD remains poorly defined, especially in adult populations. Available evidence is scarce and heterogeneous, consisting mainly of case reports, small retrospective series, and two registry-based analysis from the Extracorporeal Life Support Organization (ELSO), predominantly involving pediatric or young adult populations [9,10]. Reported outcomes are conflicting, with registry data suggesting mortality rates comparable to the general ECMO population [9,10], while adult retrospective studies—particularly in ACS—indicate substantially higher mortality [11,12]. Given these discrepancies and the absence of consolidated evidence, the clinical benefit and risks of ECMO in SCD remain uncertain, especially across different age groups, ECMO modalities, and clinical presentations.

Therefore, we conducted a systematic review and meta-analysis to synthesize the available literature on ECMO use in patients with SCD. Our objective was to estimate mortality according to ECMO modality, patient age, and the presence of ACS at cannulation.

## Methods

### Protocol and registration

The systematic review and meta-analysis was conducted in accordance with the Preferred Reporting Items for Systematic Reviews and Meta-Analyses (PRISMA) guidelines [13] and was prospectively registered in the PROSPERO database (registration number: 1120200).

### Search strategy

We performed a comprehensive search of MEDLINE, EMBASE, Pubmed and the Cochrane Central Register of Controlled Trials (CENTRAL) from January 2000 to January 2025 to identify studies reporting the use of extracorporeal membrane oxygenation (ECMO) in patients with sickle cell disease (SCD) admitted to a critical care setting, regardless of ECMO indication.

The search strategy combined controlled vocabulary and free-text terms related to ECMO (“extracorporeal membrane oxygenation,” “ECMO”), sickle cell disease (“sickle cell disease,” “SCD”), and severe respiratory complications (“acute chest syndrome,” “acute respiratory distress syndrome,” “ARDS”). Reference lists of included articles were manually screened to identify additional relevant studies. Conference abstracts were excluded, and duplicate records were removed prior to screening, in accordance with PRISMA recommendations [14]. All potential eligible papers were retrieved in full.

### Study selection and eligibility criteria

We included observational studies reporting outcomes of ECMO in patients with confirmed SCD, regardless of age, ECMO modality, or clinical indication. Studies involving patients with sickle cell trait only, or with an inclusion period prior to 1998, were excluded [15]. Case reports were excluded from the quantitative synthesis but retained for narrative synthesis because of the rarity of ECMO use in this population.

### Data extraction

Two independent reviewers screened titles and abstracts, followed by full-text assessment of potentially eligible studies. Discrepancies were resolved by consensus or adjudication by a third reviewer. Data were extracted using a standardized electronic data collection form. Data extraction was performed by one reviewer and independently verified by a second reviewer. Extracted variables included study characteristics (design, year of publication), patient demographics (age, sex, genotype when available), ECMO characteristics (veno-venous, veno-arterial, veno-arterial-venous, or central ECMO), ECMO indication, duration, and pre-cannulation clinical features (acute chest syndrome, vaso-occlusive crisis, cardiac arrest, transfusion status). Outcomes included mortality and ECMO-related complications (neurologic, thrombotic, hemorrhagic, and hemolytic events). Multiple hemorrhagic complications occurring in the same patient were counted as a single event. When necessary, corresponding authors were contacted to obtain missing data. All individual data and studies are summarized in table. There were no missing data on ECMO mode, adult vs. children, presence of ACS at cannulation and mortality. More detailed individual data lacked variably among studies, limiting its interpretation.

### Risk of bias assessment

Risk of bias for included studies was assessed using the ROBINS-I tool. All studies were retrospective observational designs and were judged to be at serious overall risk of bias, primarily due to confounding and selection bias. These limitations were considered in the interpretation of pooled estimates. The overall certainty of evidence was assessed qualitatively, considering study design, risk of bias, consistency, and precision of results.

### Statistical analysis

Meta-analyses of proportions were performed using random-effects models to account for between-study heterogeneity. Mortality proportions were logit-transformed prior to pooling and back-transformed for interpretation. Between-study heterogeneity was assessed using the I² statistic. Publication bias was explored using funnel plots and Egger’s regression test, acknowledging the limited power of these methods given the small number of included studies.

### Meta-regression

Exploratory random-effects meta-regression analyses were conducted to examine the association between mortality and prespecified study-level moderators, including the proportion of adult patients, the proportion of veno-venous ECMO, and the proportion of patients with acute chest syndrome at cannulation. Each moderator was analyzed in a separate univariable model due to limited statistical power and collinearity. Predicted mortality estimates were derived across the observed range of each moderator and uncertainty was assessed using nonparametric bootstrap resampling (2,000 iterations). All analyses were performed using R statistical software (R 3.4.4) and the metafor package.

## Results

### Study selection

The database search identified 35 records from MEDLINE, 154 from EMBASE, 35 from PubMed, and none from the Cochrane Central Register of Controlled Trials. After removal of 61 duplicates, 132 records were excluded following title and abstract screening. One registry study from the Extracorporeal Life Support Organization (ELSO) was excluded due to overlap with a more recent registry analysis and inclusion of patients prior to 2000 [9]. One case report was excluded because it involved a patient with sickle cell trait rather than sickle cell disease. Overall, 29 studies met inclusion criteria, including 7 retrospective observational studies included in the meta-analysis and 22 case reports included in the narrative synthesis only (Fig. 1).

**Fig. 1:**
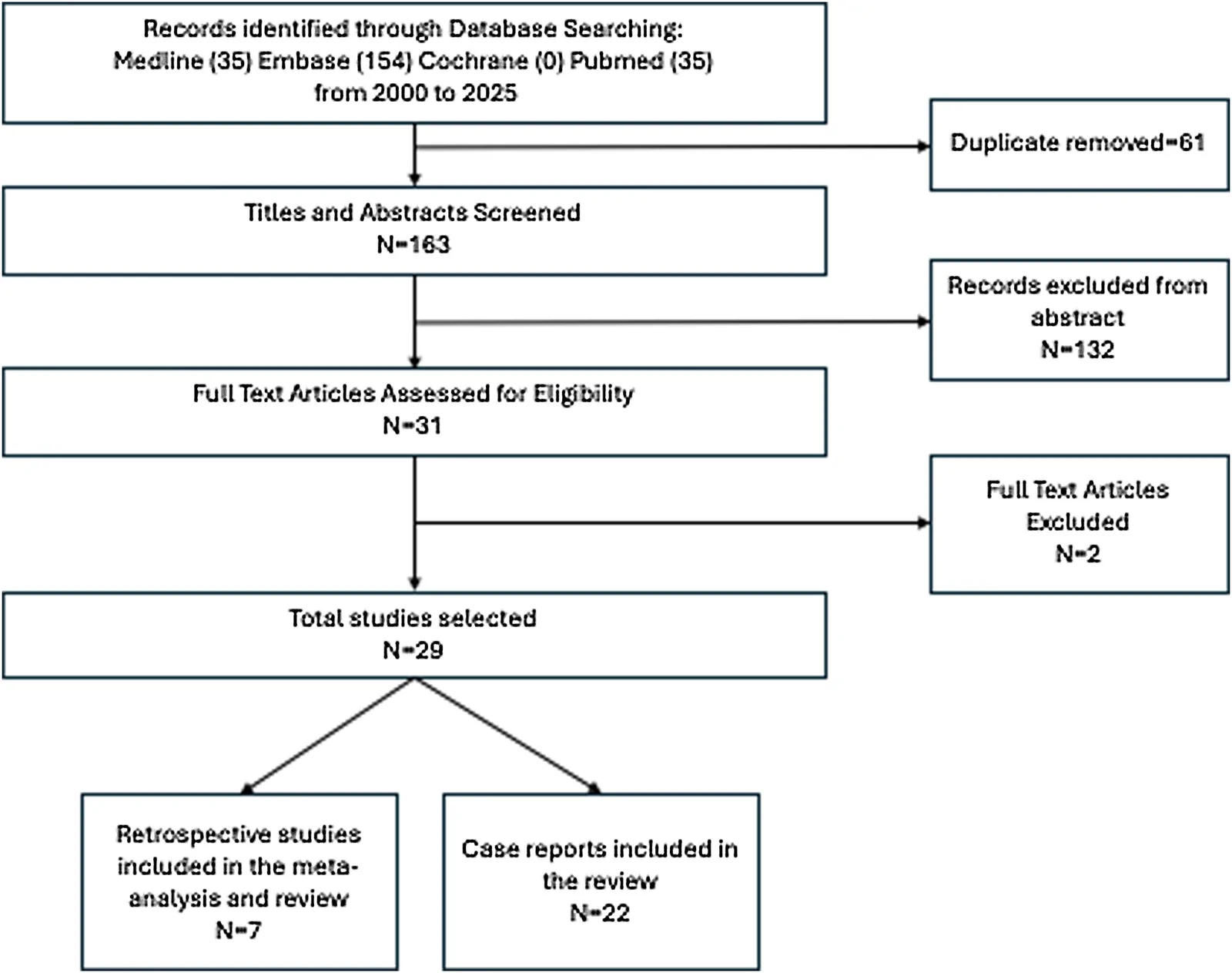
Study selection flow diagram. Flow diagram of the study selection process. A total of 224 records were identified through database searching (MEDLINE, n = 35; Embase, n = 154; Cochrane Library, n = 0, Pubmed, *n* = 35) covering the period from 2000 to 2025. After removal of duplicates (n = 61), 163 records were screened based on titles and abstracts, of which 132 were excluded. Thirty-one full-text articles were assessed for eligibility, and two were excluded after full-text review. Ultimately, 29 studies were included in the systematic review, comprising seven retrospective observational studies included in the meta-analysis and 22 case reports included in the qualitative synthesis.

### Study characteristics

The 7 retrospective studies included a total of 252 patients supported with ECMO [[10], [11], [12],[7], [16], [17], [18]]. The median age across studies was 24.5 years, and 67% of patients had homozygous SS genotype when reported (Table 2). Most patients were included after 2013 (83%). Acute chest syndrome was the indication for ECMO in 42% of cases, while extracorporeal cardiopulmonary resuscitation accounted for 14%. Veno-venous ECMO was used in 45% of patients and veno-arterial ECMO in 55%. Median ECMO duration was 5 days. Among patients with available data, RV failure was reported in 27/39 (69%), pulmonary embolism in 5/17 (29%), and multiorgan failure in 23/39 (59%). Mortality in those subgroups were not assessed because of the lack of individual data. Reported complications included thrombotic events in 22 patients (9%), excluding intra-cranial ischemia. Neurologic complications occurred in 31 patients (14%), including 17 hemorrhagic and 15 ischemic events; one patient experienced both types of events. Neurologic complications were associated with 13 reported cases of brain death (Supplemental Table S2) Fifty-seven patients (24%) experienced at least one hemorrhagic complication (excluding intra-cranial hemorrhage) with a total of 79 hemorrhagic complications recorded among those patients Hemolysis was specifically reported in one study, which described increased peak lactate dehydrogenase levels during ECMO support. A total of 83 patients (35%) required renal replacement therapy (RRT). Overall, 122 of 252 patients (48%) died during ECMO support. Cardiac arrest prior to cannulation was reported in 85 of 239 patients, and 22 of 247 patients experienced cardiac arrest during ECMO support.

The 22 case reports described 22 patients with a median age of 21 years, predominantly with SS genotype (94%) [9,[19], [20], [21], [22], [23], [24], [25], [26], [27], [28], [29], [30], [31], [32]]. Eighty-two percent of cases occurred after 2013. Acute chest syndrome was the indication for ECMO in 73% of cases. Veno-venous ECMO was used in 55% and veno-arterial ECMO in 36% of cases. Overall mortality in case reports was 18% (Table 1, Supplemental Table S1).

**Table 1:** Characteristics of retrospective studies included in the meta-analysis and systematic review.

Study ID	Author	Study period	Study type	Number of patients included per study	Age (median, years)	Genotype HbSS n (%)	ECMO VV n (%)	ECMO VA n (%)	ACS at ECMO cannulation n (%)	Mortality n (%)
1	Cecchini J.	2004−2010	Retrospective	3	22	2 (67%)	3 (100%)	0 (-)	3 (100%)	3 (100%)
2	Boissier F.	2009−2017	Retrospective	22	27	15 (68%)	10 (45%)	12 (55%)	22 (100%)	16 (73%)
3	Alashkar F.	2017−2018	Retrospective	3	20	2 (67%)	3 (100%)	0 (-)	3 (100%)	0 (-)
4	Abgakou M.	2015−2017	Retrospective	8	28.5	NA (-)	5 (63%)	3 (38%)	3 (38%)	5 (63%)
5	Ilerhunmwuwa NP.	2023	Retrospective	5	NA	4 (80%)	5 (100%)	0 (-)	0 (-)	3 (60%)
6	Rémy T.	1998−2022	Retrospective (ELSO registry)	208	17.6	NA (-)	88 (42%)	120 (58%)	73 (35%)	92 (44%)
7	Grazioli A.	2024	Retrospective	3	48	1 (33%)	0 (-)	3 (100%)	3 (100%)	3 (100%)

The table summarizes study design and period, number of patients included, patient age, sickle cell genotype distribution, extracorporeal membrane oxygenation configuration (veno-venous [VV] or veno-arterial [VA]), presence of acute chest syndrome at the time of ECMO cannulation, and in-hospital mortality. Data are presented as median values or number (percentage), as reported in the original studies. Missing data are indicated as not available.

**Table 2:** Individual clinical characteristics and ECMO-related data from included studies.

	Retrospective studies, N = 252	Case reports, N = 22
**Age**, years, median, [IQR 25-75], N	24 [21–28], N = 247	21 [18–29], N = 22
**Inclusion period:**		
Before 2013	38/230 (17%)	4/22 (18%)
After 2013	192/230 (83%)	18/22 (82%)
**SCD genotype**, n/N (%)		
SS	24/36 (67%)	16/17 (94%)
SC	7/36 (19%)	1/17 (6%)
Sβ-thalassemia	4/36 (11%)	0/17 (0%)
Other SCD genotypes	1/36 (3%)	0/17 (0%)
**Recent VOC prior to ECMO**, n/N (%)	7/9 (78%)	11/21 (52%)
**Red blood cell exchange or transfusion prior to ECMO**, n/N (%)	20/31 (65%)	15/21 (71%)
**Hydroxycarbamide prior to ECMO**, n/N (%)	14/25 (56%)	2/21 (10%)
**Allo-immunization**, n/N (%)	6/28 (21%)	3/20 (15%)
**Duration of hospital stay prior to ECMO**, days, median, [IQR 25-75], N	2.5 [2–4], N = 31	3 [1–5], N = 16
**Duration of invasive mechanical ventilation prior to ECMO**, days, median [IQR 25-75], N	1 [1–1], N = 28	0.5 [0−2], N = 18
**ECMO indication**, n/N (%)		
ACS	107/252 (42%)	16/22 (73%)
E-CPR	34/239 (14%)	1/22 (5%)
**Associated organ failure**, n/N (%)		
LV failure	15/39 (38%)	8/22 (36%)
RV failure / acute cor pulmonale	27/39 (69%)	13/22 (59%)
Pulmonary embolism	5/17 (29%)	2/22 (9%)
Documented fat embolism	3/17 (18%)	2/22 (9%)
Renal failure	4/17 (24%)	7/21 (33%)
Renal replacement therapy	83/236 (35%)	6/22 (27%)
Multi-organ failure	23/39 (59%)	9/21 (43%)
**ECMO mode**, n/N (%)		
VV	114/252 (45%)	12/22 (55%)
VA	138/252 (55%)	8/22 (36%)
Other	0/252 (0%)	1/22 (5%)
**ECMO duration**, days, median, [IQR 25-75], N	5 [4–6], N = 236	7 [5–12], N = 21
**ECMO complications**, n/N (%)		
Hemorrhagic*	57/236 (24%)	0/22 (0%)
Thrombotic**	22/236 (9%)	2/22 (9%)
Neurologic	31/214 (14%)	4/22 (18%)
Hemolysis	NR	1/22 (5%)
**Cardiac arrest under ECMO**, n/N (%)	22/247 (9%)	5/22 (23%)
**Cardiac arrest before ECMO**, n/N (%)	85/239 (36%)	5/22 (23%)
**Mortality**, n/N (%)	122/252 (48%)	4/22 (18%)

ACS: acute chest syndrome, ECMO: extracorporeal membrane oxygenation, E-CPR: extracorporeal cardiopulmonary resuscitation, LV: left ventricle, NR: not reported, RV: right ventricle, SCD: sickle cell disease, VA: veno-arterial, VOC: vaso-occlusive crisis, VV: veno-venous.

*Hemorrhagic complications exclude intra-cranial bleeds.

**Thrombotic complications exclude intra-cranial ischemia.

Individual clinical characteristics and ECMO-related data from included studies stratified by retrospective studies and case reports. The table summarizes patient demographics, sickle cell disease (SCD) genotype distribution, clinical status before ECMO initiation, ECMO indications and configuration, associated organ failures, ECMO duration, complications, cardiac arrest occurrence, and in-hospital mortality. Data are reported as median values [IQR] or number (percentage), according to availability in the original studies. Denominators correspond to available data for each variable and may differ from total sample size, when data were not available for all patients. Missing data are indicated where applicable.

### Pooled mortality

Meta-analysis of the 7 retrospective studies yielded a pooled mortality estimate of 64.5% (95% CI, 39.0–86.0%) using a random-effects model (Fig. 2). Between-study heterogeneity was substantial (I² = 80%, τ² = 0.0785; p < 0.01).

**Fig. 2:**
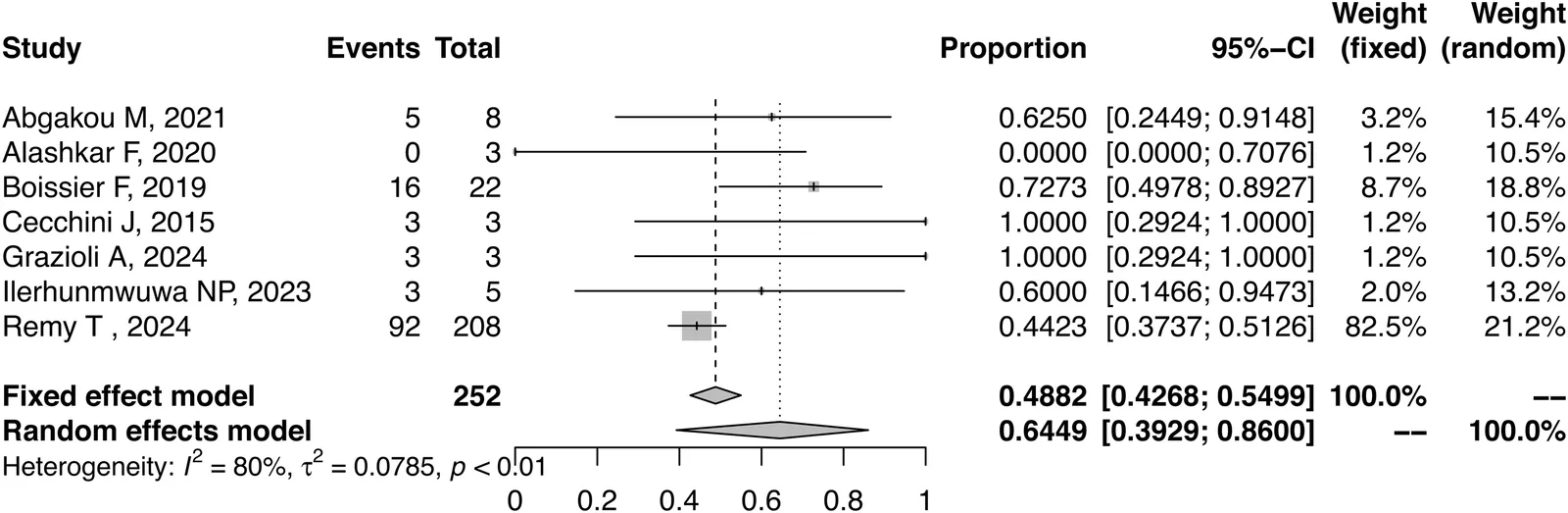
Forest plot of pooled mortality in patients with sickle cell disease (SCD) supported by extracorporeal membrane oxygenation (ECMO). Forest plot of mortality in patients with sickle cell disease (SCD) receiving extracorporeal membrane oxygenation (ECMO). Study-specific mortality proportions with corresponding 95% confidence intervals are shown. The pooled estimate was derived using a random-effects model for proportions, with study weights based on inverse variance. Statistical heterogeneity across studies was quantified using the I² statistic.

### Subgroup analyses

When stratified by age, mortality was higher in adults (73.0%, 95% CI: 50.4–90.6%) compared with children under 18 years (53.0%, 95% CI: 43.2–62.6%) (Table 3). Regarding ECMO modality, mortality reached 83.3% (95% CI: 35.9–99.6%) for patients supported with veno-arterial ECMO (VA-ECMO), compared with 54.5% (95% CI: 23.4–83.3%) for those under veno-venous ECMO (VV-ECMO). Mortality was also slightly higher among patients with acute chest syndrome (ACS) (66.7%, 95% CI: 39.1–86.2%) than among those without ACS (60.0%, 95% CI: 22.9–88.4%).

**Table 3:** Pooled mortality estimates by patient and ECMO characteristics.

	Mortality	
	Pooled incidence (%)	Confidence interval 95%
Overall	64.5	[39.3−86.0]
Adult	73.0	[50.4−90.6]
Children (<18yo)	53.0	[43.2−62.6]
ECMO VV	54.5	[23.4−83.3]
ECMO VA	83.3	[35.9−99.6]
ACS	66.7	[39.1−86.2]
No ACS	60.0	[22.9−88.4]

ACS, acute chest syndrome, ECMO, extracorporeal membrane oxygenation; VA, veno-arterial; VV, veno-venous.

Pooled mortality estimates derived from random-effects meta-analyses of proportions, stratified according to predefined study-level and patient characteristics, including age group, extracorporeal membrane oxygenation (ECMO) configuration, and presence of acute chest syndrome (ACS). Estimates are presented as pooled percentages with corresponding 95% confidence intervals. Subgroup analyses were exploratory and based on available aggregated data from the included retrospective studies.

Note: Pooled estimates were calculated using random-effects models. Subgroup analyses were not adjusted for potential confounders and should be interpreted with caution.

### Meta-regression

Meta-regression demonstrated a positive association between the proportion of adults and predicted mortality under ECMO (Fig. 3A). Predicted mortality increased approximately linearly with the proportion of adults, ranging from ∼30% in fully pediatric cohorts to nearly 70% in fully adult cohorts. Higher proportions of venovenous ECMO had lower predicted mortality, decreasing from approximately 0.70 to 0.50 across the range of VV use (Fig. 3B).

**Fig. 3:**
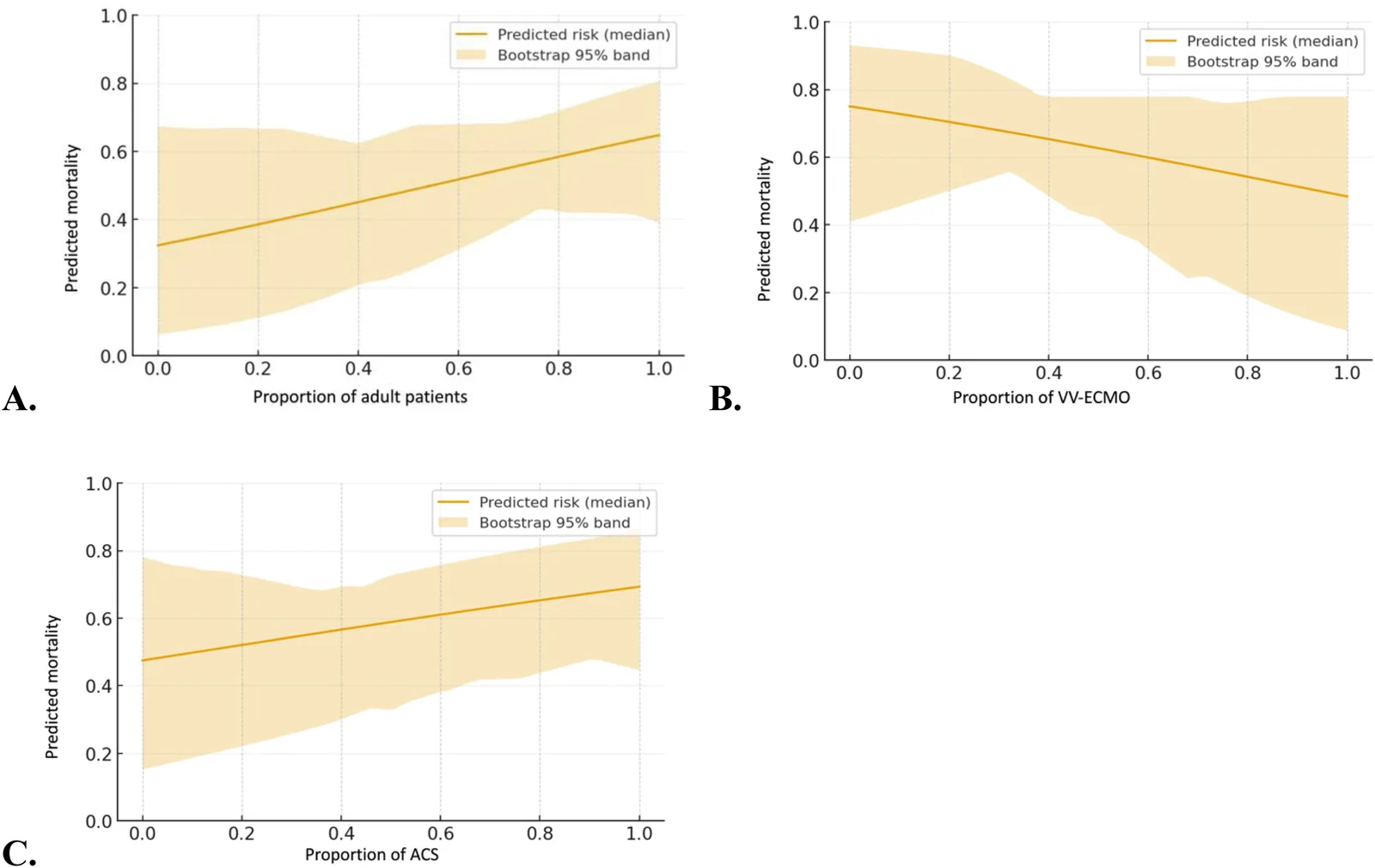
Marginal effects of study-level covariates on predicted mortality from meta-regression analyses. Marginal effects from meta-regression analyses showing the association between study-level covariates and predicted mortality among patients with sickle cell disease supported by extracorporeal membrane oxygenation. Solid lines represent the predicted mortality based on the fitted meta-regression model, and shaded areas indicate 95% bootstrap confidence bands derived from resampling at the study level. (A) Proportion of adult patients. (B) Proportion of patients supported with veno-venous extracorporeal membrane oxygenation (ECMO-VV). (C) Proportion of patients with acute chest syndrome (ACS) as the primary indication.

Increasing proportions of patients with acute chest syndrome were associated with higher predicted mortality. Predicted values rose from about 0.45 to nearly 0.70 across the ACS gradient (Fig. 3C).

### Risk of bias and publication bias

Visual inspection of funnel plots did not reveal clear asymmetry, and Egger’s regression test did not provide strong evidence of small-study effects. However, the limited number of studies precluded reliable assessment of publication bias. Using the ROBINS-I tool [33], all included studies were judged to be at serious overall risk of bias, primarily due to confounding and selection bias inherent to retrospective observational designs (Supplemental Fig. S1).

## Discussion

In this systematic review and meta-analysis, we provide the first pooled estimate of mortality among adult and pediatric patients with sickle cell disease (SCD) supported with extracorporeal membrane oxygenation (ECMO). Across seven retrospective cohorts encompassing 252 patients, pooled mortality was estimated at 64.5%, with substantial between-study heterogeneity (95%-CI of [39–86 %]). This estimate appears higher than mortality reported in registry-based analyses of younger and more heterogeneous SCD populations from the Extracorporeal Life Support Organization (ELSO), highlighting important differences in case mix and clinical context. Indeed, Rémy et. al described a global mortality of 44% in a SCD population including 208 patients from age 0 to 40 years old from the ELSO registry before 2024 [10]. In a previous ELSO registry study by Kuo et. al, that looked at a pediatric population (from 30 days to 18 years old) included in the registry between 1990 and 2012, the overall mortality was 48% [9].

The study population was heterogeneous, encompassing a broad age range and diverse indications for ECMO support. Many of the included patients were supported for indications other than acute chest syndrome (ACS), from post-operative, to congenital heart diseases in infants for VA-ECMO or other causes of ARDS than ACS for VV-ECMO. In contrast, most non-registry cohorts included in the meta-analysis focused on critically ill older children and adults, frequently with severe ACS and multiorgan failure, which likely contributes to the higher observed mortality [17]. In the present analysis, ACS accounted for approximately 42% of ECMO indications, which resulted in a pooled mortality of 66.7%. Exploratory meta-regression suggested a trend toward increasing mortality with increasing proportions of ACS, although uncertainty widened at the extremes. These findings should be interpreted cautiously but are biologically plausible. Unlike infectious ARDS, ACS reflects the convergence of multiple injurious mechanisms—including vaso-occlusion, thrombosis, infection, fat embolism, and hemolysis—often leading to pulmonary hypertension, right ventricular failure, and multiorgan dysfunction [6]. Such complexity may limit the effectiveness of ECMO as an isolated rescue strategy.

Another potential source of heterogeneity relates to the temporal evolution of ECMO practices. ECMO technology, cannulation strategies, patient selection, and critical care management have substantially evolved over the past two decades. A temporal analysis might therefore have helped determine whether the observed mortality was partly attributable to historical differences in ECMO management rather than to disease severity alone. However, such an analysis could not be reliably performed because individual patient-level data were unavailable for three of the seven included studies. Notably, 83% of the patients in our meta-analysis were treated after 2013, suggesting that our pooled estimate predominantly reflects contemporary ECMO practice.

Mortality estimates appeared higher in studies including predominantly adult populations compared with pediatric cohorts. Exploratory analysis suggested increasing predicted mortality with increasing proportions of adult patients. This observation is consistent with the cumulative burden of cardiopulmonary and renal complications that accrues with age in SCD [[34], [35], [36]], as well as the higher mortality associated with ACS in adults, which has been reported to be approximately fourfold higher than in pediatric patients [37]. However, these findings arise from study-level analyses and cannot disentangle the effects of age from differences in disease severity, timing of cannulation, use of extracorporeal cardiopulmonary resuscitation, or transfusion practices.

When looking at ECMO modality, the pooled mortality estimate was 83.3% in the VA-ECMO group compared to 54.5% in the VV-ECMO group. In this setting, the need for VA-ECMO likely reflects extreme disease severity rather than a harmful effect of the modality itself. In SCD, VA-ECMO is often instituted in the context of profound circulatory failure, frequently driven by acute pulmonary hypertension and right ventricular dysfunction as reflected in the meta-analysis (69% of patients had right ventricular (RV) failure, 29% had a confirmed pulmonary embolism and 59% had associated multi-organ failure). The lack of individual patient-level data limited our ability to differentiate causes of mortality in VA-ECMO patients. Notably, Boissier et al. observed increased mortality in patients with multi-organ failure, while patients with isolated right ventricular failure demonstrated more favorable outcomes following VA-ECMO support [7]. For VV-ECMO, the pooled mortality estimate in our SCD population was higher than mortality reported in the EOLIA and CESAR trials (35% and 37%, respectively), although direct comparisons are limited by major differences in patient populations and underlying diseases [38,39].

From a clinical perspective, patients with SCD requiring ECMO should be managed in centers with expertise in both ECMO and SCD. Prompt and optimized treatment of ACS and acute hemolysis, including transfusion or exchange transfusion when appropriate, should be prioritized before and during ECMO support, as severe ACS and hemolysis may contribute to multiorgan dysfunction and fat embolism, potentially compromising outcomes despite extracorporeal support [40]. In SCD patients, refractory hypoxemia or acute right ventricular dysfunction despite optimized conventional management may prompt consideration of ECMO. In some cases of refractory acute cor pulmonale despite ECMO, conversion to veno-pulmonary arterial assist could be a salvage therapy [41].

Reporting of ECMO-related complications was inconsistent across studies. Rates of hemorrhagic, thrombotic, and neurologic events did not appear markedly higher than those reported in large ECMO cohorts, although underreporting is likely. Hemolysis, a key concern in SCD, was rigorously assessed in only one study, which reported universal elevations in lactate dehydrogenase during ECMO support [7]. The interaction between ECMO-associated shear stress, hemolysis, and microvascular obstruction remains poorly characterized and warrants focused investigation.

This study is limited by the small number of retrospective observational studies, substantial heterogeneity, long inclusion period, inconsistent reporting, and wide confidence intervals, particularly in subgroup analyses. In addition, all studies were considered to have a high risk of bias. The absence of individual-level data precluded adjustment for baseline severity, timing of cannulation, and co-interventions. Meta-regression analyses were exploratory and subject to ecological bias and collinearity [42]. Case reports introduce publication bias and were included only for narrative synthesis.

Despite these limitations, this synthesis underscores the high mortality associated with ECMO in patients with SCD, particularly in severe presentations such as ACS. These findings highlight the urgent need for prospective, disease-specific registries with standardized reporting of severity, timing and indication of cannulation, transfusion strategies, anticoagulation, and hemolysis markers.

## Author contributions

LF: Conceptualization, Literature search, Study selection, Data curation, Investigation, Writing - original draft.

AR: Conceptualization, Methodology, Formal analysis, Validation, Writing - review & editing.

EV: Investigation, Data curation, Writing - review & editing.

TP: Investigation, Data curation, Writing - review & editing.

All authors contributed to data interpretation, critically reviewed the manuscript, and approved the final version for publication.

## Consent for publication

Not applicable.

## Ethical approval and consent to participate

Not applicable. This systematic review and meta-analysis used data from previously published studies and did not involve individual patient data.

## Funding

This research received no external funding

## Availability of supporting data

The dataset generated is available from the corresponding author upon reasonable request. All included studies are cited in the manuscript.

## Declaration of competing interest

No conflicts of interest to declare.
