# Supplementary material for: Outcomes of extracorporeal membrane oxygenation in critically ill patients with sickle cell disease: a systematic review and meta-analysis
Source: Ann Intensive Care. 2026 Oct 28;16:100194. doi: 10.1016/j.aicoj.2026.100194 (PMC13761238; doi:10.1016/j.aicoj.2026.100194)
Supplement: Supplementary file 1 [file mmc1.docx]

**Supplemental figure and table legends**

**Supplemental Figure 1. Risk of bias**

**Supplemental Figure 2. Funnel plot**

**Supplemental Table 1. Case report characteristics**

**Supplemental Table 2. Reported ECMO Complications**

**Supplemental Figure 1. Risk of bias assessment using the ROBINS-I tool**

**Supplemental Figure 1.** **Risk of bias assessment of the included retrospective studies using the ROBINS-I (Risk Of Bias In Non-randomized Studies of Interventions) tool.** Each row represents an individual study and each column corresponds to one of the seven ROBINS-I domains: bias due to confounding (D1), selection of participants (D2), classification of interventions (D3), deviations from intended interventions (D4), missing data (D5), measurement of outcomes (D6), and selection of the reported result (D7). The overall risk of bias for each study is shown in the final column and reflects the highest risk judgment across domains, in accordance with ROBINS-I guidance. Risk levels are indicated by color coding: low (green), moderate (yellow), serious (orange), and critical (red).

**Supplemental Figure 2. Funnel plot**

**Supplemental Figure 2. Funnel plot of study-specific mortality estimates plotted against their standard errors for the meta-analysis of patients with sickle cell disease supported by extracorporeal membrane oxygenation.** The vertical dashed line represents the pooled mortality estimate derived from the random-effects model, and diagonal dashed lines indicate the expected 95% confidence limits in the absence of small-study effects. Visual inspection of the funnel plot was used to explore publication bias and small-study effects

**Supplemental Table 1. Case report characteristics**

| **Authors** | **Country** | **journal** | **Year of publication** | **Number of patients** | **Age (years, median)** | **Phenotype** | **ECMO** | **Summary** | **ACS** | **ECMO duration**  **(days)** | **Complications** | **Cardiac arrest (n=)** | **Death (n=)** |
| --- | --- | --- | --- | --- | --- | --- | --- | --- | --- | --- | --- | --- | --- |
| Grotberg J.C. | USA, MO | Artificial organs | 2024 | 1 | 25 | SS | VV | Context: ACS complicated by severe ARDS and RV dilatation requiring VV-ECMO at day HD6.  TTE: RV dilatation (5cm) with preserved systolic function  Treatment received: Antibiotics, RBC exchange transfusion (HBS 77% to 23%), mechanical ventilation, paralysis, proning, epoprostenol. No heparin due to persistent thrombocytopenia.  Complication: hemolysis (improved after reducing ECMO flow to 4.5L/min) | Yes | 18 | Hemolysis | 0 | 0 |
| Nicholls J.R. | UK, Manchester | Journal of Cardiothoracic and Vascular Anesthesia | 2022 | 1 | 18 | SS | VV | Context: VOC with chest and leg location progressed to ACS and complicated with ARDS with suspected fat emboli, and multi-organ failure requiring VV-ECMO.  TTE: signs of vasoplegia, normal LV function, normal RV function, normal pulmonary artery systolic pressure  Treatment received: RBC exchange transfusion, mechanical ventilation, noradrenaline, hydrocortisone, RRT, plasma exchange. Heparin 1,000UI/h throughout ECMO run.  Complication of ECMO: none | Yes | 7 | 0 | 0 | 0 |
| Koh W. | USA, OH | Pediatric pulmonology | 2022 | 1 | 11 | SS | VV | Context: ACS related to COVID-19 complicated by severe ARDS requiring VV-ECMO.  TTE: normal RV pressure and function  Treatment received: steroids, convalescent plasma, remdesivir. Mechanical ventilation, proning, inhaled nitric oxide, RBC exchange transfusion, hydroxyurea, plasma and RBC transfusion. Anticoagulation (not specified).  Complication of ECMO: none. Discharged home 5 months after initial presentation with mechanical ventilation (tracheostomy). | Yes | 71 | 0 | 0 | 0 |
| Teulier M. | France, Paris | BMC pulmonary Med | 2021 | 1 | 33 | SS | VV | Context: COVID-19 complicated by severe ARDS requiring VV-ECMO.  TTE: normal LV and RV functions  Treatment received: RBC exchange transfusion, broad-spectrum antibiotics, mechanical ventilation, paralysis, proning. Anticoagulation (therapeutic dosage).  Complication of ECMO: vena-cava inferior thrombosis | No | 10 | Thrombosis | 0 | 0 |
| Tanenbaum MT | USA, VA | The American Surgeon ET blood | 2020 | 1 | 32 | NA | VAV | Context: ACS complicated by ARDS leading to hypoxic cardiac arrest with multi-organ failure, refractory ventricular fibrillation requiring E-CPR with VA ECMO, transitioned to a VAV-ECMO due to severe hypoxia.  TTE: normal LV function, RV dilatation and overload (RV function not specified).  Treatment received: broad-spectrum antibiotics, mechanical ventilation, norepinephrine, epinephrine, phenylephrine, dobutamine, RRT, RBC exchange transfusion.  Complication of ECMO: Grey-white inversion with permanent brain damage and vegetative state. | Yes | 28 | Permanent brain damage | 1 | 1 |
| de Roux Q | France, Paris | The Annals of Thoracic Surgery | 2020 | 1 | 35 | SS | VA | Context: Cardiogenic shock with multi-organ failure due to bioprosthetic aortic and mitral valve degeneration requiring valve replacement surgery, complicated with severe biventricular dysfunction at bypass weaning requiring VA-ECMO.  TTE: LV function 40%, pulmonary systolic arterial pressure 75mmHg, moderate tricuspid regurgitation.  Treatment received: diuretics, dobutamine, norepinephrine, epinephrine, RBC exchange transfusion, RBC transfusion, RRT, heparin  Complication of ECMO: cannula site infection (femoral) | 0 | 6 | Cannula site infection | 1 | 0 |
| Al-Sawaf O | Germany, Cologne | Annals of Hematology | 2019 | 1 | 21 | SS | VV | Context: VOC progressed to ACS due to VRS and Chlamydia pneumoniae complicated with ARDS requiring VV-ECMO.  TTE: LV function 20%  Treatment received: Antibiotics, mechanical ventilation, noradrenaline, dobutamine, RRT, RBC exchange transfusion.  Complication of ECMO: none | 0 | 7 | 0 | 0 | 0 |
| Le Guennec, L | France, Paris | Intensive Care Medicine | 2019 | 1 | 29 | NA | VA | Context: VOC progressed to ACS and ARDS with acute pulmonary hypertension requiring VA-ECMO.  TTE: acute pulmonary hypertension  Treatment received: NA  Complication of ECMO: neurologic reversible splenial lesion syndrome | 1 | 5 | Reversible splenial lesion syndrome | 0 | 0 |
| Sewaralthahab SS | USA, MD | Hemoglobin | 2018 | 1 | 25 | SS | VV | Context: ACS progressed to ARDS requiring VV-ECMO  TTE: preserved LV and RV function, mild pulmonary hypertension  Treatment received: antibiotics, mechanical ventilation, paralysis, proning, RBC exchange transfusion, RBC transfusion, heparin.  Complication of ECMO: Deep vein thrombosis at cannulation sites, pneumothorax | 1 | 20 | Thrombosis, pneumothorax | 0 | 0 |
| Mashhour A | Germany, Oldenburg | Artificial organs | 2017 | 1 | 14 | NA | VV | Context: ACS progressed to ARDS requiring VV-ECMO  TTE: NA  Treatment received: NA  Complication of ECMO: none | 1 | 5 | 0 | 0 | 0 |
| Wassim BA | Tunisie | La Tunisie médicale | 2016 | 1 | 7 | SS | Central | Context: Urgent aortic and mitral replacement for acute endocarditis complicated with biventricular dysfunction requiring central ECMO.  TTE: LV function 73%, aortic and mitral vegetations with regurgitation (grade III), RV dysfunction with tricuspid insufficiency and pulmonary hypertension  Treatment received: no exchange transfusion before surgery, blood transfusion during surgery, heparin, adrenalin, mechanical ventilation. Died at H48 of multiorgan failure.  Complication of ECMO: none | 0 | 2 | 0 | 1 | 1 |
| Parhar K | UK, Cambridge | Perfusion | 2015 | 1 | 18 | SS | VV | Context: Salmonella non-typhi infection complicated with ACS and ARDS requiring VV-ECMO.  TTE: NA  Treatment received: mechanical ventilation, exchange transfusion, IV immunoglobulin due to allo-immunization, antibiotics, heparin.  Complication of ECMO: none | 1 | 12 | 0 | 0 | 0 |
| Kuo KW | USA, MI | Perfusion | 2013 | 1 | 6 | SS | VA | Context: ACS complicated with biventricular dysfunction requiring VA-ECMO.  TTE: biventricular dysfunction, LV function 20%, RV dysfunction with severe tricuspid regurgitation.  Treatment received: mechanical ventilation, red blood transfusion, epinephrin, milrinone, antibiotics, heparin.  Complication of ECMO: massive brain hemorrhage requiring craniectomy. | 0 | 7 | Massive brain hemorrhage | 0 | 0 |
| Grein E | France, Nancy | Annales Francaises d’Anesthésie et de Réanimation | 2012 | 1 | 21 | SC | VA | Context: ACS complicated with ARDS, pulmonary embolism and RV dysfunction (acute cor pulmonale) requiring VA-ECMO.  TTE: RV dysfunction with pulmonary hypertension  Treatment received: thrombolysis, mechanical ventilation, antibiotics, blood exchange transfusion, dobutamine, noradrenaline, renal replacement therapy.  Complication of ECMO: none | 1 | 11 | 0 | 1 | 0 |
| Hoffman M | Germany | DMW | 2011 | 1 | 28 | SS | VV | Context: ACS complicated with refractory ARDS requiring VV-ECMO.  TTE: NA  Treatment received: mechanical ventilation, blood exchange transfusion, antibiotics.  Complication of ECMO: none | 1 | 12 | 0 | 0 | 0 |
| Stendahl G | USA, WI | Progress in transplantation | 2010 | 1 | 10 | SS | VA | Context: Humoral rejection with biventricular dysfunction 2 years after heart transplantation, requiring VA-ECMO.  TTE: Severe biventricular dysfunction  Treatment received: mechanical ventilation, epinephrin, norepinephrine, vasopressin, renal replacement therapy, thymoglobulin, mycophenolate, and steroids.  Complication of ECMO: none | 0 | 5 | 0 | 0 | 0 |
| Avgeridou S | Germany, Cologne | SN Comprehensive Clinical Medicine | 2021 | 1 | 19 | SS | VA | Context:ACS complicated with refractory ARDS and massive pulmonary embolism with acute cor pulmonale requiring VA-ECMO.  TTE: severe RV dysfunction and acute core pulmonale  Treatment received: antibiotics, thrombolysis, anticoagulation, mechanical ventilation after cannulation, thrombectomy.  Complication of ECMO: none | 1 | 6 | 0 | 0 | 0 |
| Belveyre T | France, Nancy | Respiratory Medicine Case Reports | 2019 | 1 | 19 | SS | VV | Context: ACS complicated with refractory ARDS and acute core pulmonale  TTE: LV function preserved, RV failure with pulmonary hypertension  Treatment received: antibiotics, analgesia, red blood cell transfusion, mechanical ventilation, paralysis, inhaled nitric oxide, norepinephrine.  Complication of ECMO: none | 1 | 7 | 0 | 0 | 0 |
| Chambers J | USA, IL | Case Rep Crit Care | 2018 | 1 | 20 | NA | VV | Context: ACS at 25-week gestation complicated with refractory ARDS requiring VV-ECMO.  TTE: NA  Treatment received: antibiotics, analgesia, red blood cell exchange transfusion, mechanical ventilation.  Complication of ECMO: none | 1 | NA | 0 | 0 | 0 |
| Sandoval E | Spain, Barcelona | Perfusion | 2020 | 1 | 36 | SS | VA | Context: ACS complicated with ARDS, acute core pulmonale and biventricular dyfunction requiring VA-ECMO.  TTE: severe biventricular dyfunction  Treatment received: NA  Complication of ECMO: none | 1 | 6 | 0 | 0 | 0 |
| Shyu S | USA, MD | Human pathology: Case reports | 2017 | 1 | 48 | SS | VA | Context: ACS and fat embolism complicated with acute core pulmonale requiring VA-ECMO due to parvovirus B19. Rapidly deteriorated under ECMO with multi-organ failure and multiple cardiac arrests.  TTE: dilated RV, moderate tricuspid regurgitation.  Treatment received: mechanical ventilation, red blood cell transfusion, steroids, IV immunoglobulins, plasmapheresis, heparin.  Complication of ECMO: none | 1 | 1 | 0 | 1 | 1 |
| Livesay S | USA, TX | Critical Care Nursing Quarterly | 2012 | 1 | 24 | NA | VV | Context: ACS complicated with ARDS and acute core pulmonale requiring VV-ECMO.  TTE: Estimated left ventricular ejection fraction of  60%. Moderate tricuspid regurgitation, pulmonary hypertension (75-80 mmHg) Right atrial pressure estimated at 6-10 mm Hg.  Treatment received: analgesia, red blood cells transfusion, antibiotics, mechanical ventilation, vasopressors.  Complication of ECMO: Multifocal brain hemorrhage leading to brain death. | 1 | 2 | Brain hemmorhage | 0 | 1 |
| TOTAL | - | - | - | 22 | 21 | - | - | - | 16 (73%) | 7 (median, days) | 8 (36%) | 5 (23%) | 4 (18%) |

**Supplemental Table 2. Reported ECMO Complications**

|  | **Retrospective studies**, N=252 | **Case reports**, N=22 |
| --- | --- | --- |
| **Hemorrhagic*** | Cannulation bleeding (18), surgical site bleeding (15), gastro-intestinal bleed (9), peripheral bleed (6), disseminated intravascular coagulation (6), mediastinal bleed (4), hemorrhagic complication unspecified (9), classified as hemorrhagic with hemolysis (12) | None |
| **Thrombotic**** | ECMO circuit thrombosis (15), Hemofilter thrombosis (3), clots emboli (1), Thrombotic complication unspecified (4) | Inferior vena cave thrombosis (1), Cannula insertion site thrombosis (1) |
| **Neurologic** | Subdural hematoma (1), cerebral fat embolism and hemorrhagic petechiae (2), ischemic stroke (15), brain hemorrhage (14)  *Neurological complications resulted in 13 reported brain death* | Anoxic brain injury (1), RESLES*** (1), brain hemorrhage (2)  *Neurological complications resulted in 1 brain death and 1 vegetative state.* |
| **Hemolysis** | **Not clearly reported:**  Increased reported hemolysis in 22 patients from Boissier et al., 12 unspecified hemolysis from Rémy et. al classified under hemorrhagic complications. | Increased reported hemolysis (1) |

* Hemorrhagic events exclude intra-cranial hemorrhage. In the 7 retrospective studies, 57 patients had at least 1 hemorrhagic event, 79 hemorrhagic events occurred in 57 patients

** Thrombotic events exclude brain ischemia

***RESLES: reversible splenial lesion syndrome
